# Stereo- and Regiocontrolled Syntheses of Exomethylenic Cyclohexane β-Amino Acid Derivatives

**DOI:** 10.3390/molecules201219749

**Published:** 2015-11-27

**Authors:** Loránd Kiss, Enikő Forró, György Orsy, Renáta Ábrahámi, Ferenc Fülöp

**Affiliations:** 1 Institute of Pharmaceutical Chemistry, University of Szeged, H-6720 Szeged, Hungary; kiss.lorand@pharm.u-szeged.hu (L.K.); forro.eniko@pharm.u-szeged.hu (E.F.); Orsy.gyorgy@pharm.u-szeged.hu (G.O.); abrahami.renata@pharm.u-szeged.hu (R.Á.); 2 Stereochemistry Research Group of the Hungarian Academy of Sciences, University of Szeged, H-6720 Szeged, Eötvös 6, Hungary

**Keywords:** amino acids, selectivity, hydroxylation, Wittig reaction, icofungipen

## Abstract

Cyclohexane analogues of the antifungal icofungipen [(1*R*,2*S*)-2-amino-4-methylenecyclopentanecarboxylic acid] were selectively synthesized from unsaturated bicyclic β-lactams by transformation of the ring olefinic bond through three different regio- and stereocontrolled hydroxylation techniques, followed by hydroxy group oxidation and oxo-methylene interconversion with a phosphorane. Starting from an enantiomerically pure bicyclic β-lactam obtained by enzymatic resolution of the racemic compound, an enantiodivergent procedure led to the preparation of both dextro- and levorotatory cyclohexane analogues of icofungipen.

## 1. Introduction

**Figure 1:**
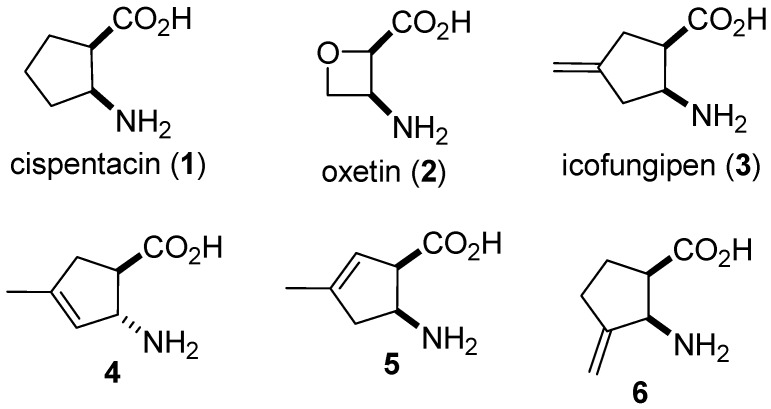
Some biologically interesting cyclic β-amino acids.

As a consequence of their high biological potential, cyclic β-amino acids are of importance in medicinal chemistry. These compounds are both elements of bioactive products and building blocks in peptide research. Several small molecular entities, such as the cyclopentane derivative cispentacin (**1**) and oxetane derivative oxetin (**2**), possess strong antifungal and antibacterial activities [1,2,3,4,5,6,7,8,9,10,11,12,13]. An exomethylene function plays an essential role in the structures of some cyclic β-amino acids. The β-amino acid (1*R*,2*S*)-2-amino-4-methylenecyclopentanecarboxylic acid (icofungipen, PLD-118, **3**) and several analogues (**4**–**6**) exhibit strong antifungal properties (Figure 1). The (−)-(1*R*,2*S*)-2-Amino-4-methylenecyclopentane carboxylic acid was analyzed by Bayer. This compound, previously known as BAY 10-8888, was licensed to Glaxo-SmithKline Research Centre Zagreb Ltd. (formerly PLIVA) and renamed PLD-118; its generic name is icofungipen. Icofungipen is a cyclic β-amino acid, which differs in chemistry, biology, and mechanism of action from other antifungal compound classes. Its mechanism of action is based on the inhibition of isoleucyl-tRNA synthetase, intracellular inhibitory concentrations at the target site being achieved by active accumulation in susceptible fungi [14,15,16,17,18].

The most efficient multigram-scale asymmetric synthetic route to icofungipen involves the asymmetric desymmetrization of the meso-anhydride of a cyclopentane exo-methylenedicarboxylic acid. In the key step, highly enantioselective, quinine-mediated alcoholysis of the *meso*-anhydride, followed by Curtius rearrangement and Pd-catalyzed removal of the protecting groups affords icofungipen (absolute configurations 1*R*,2*S*) with *ee* = 99.5% [14,15,16,17,18].

## 2. Results and Discussion

**Scheme 1:**
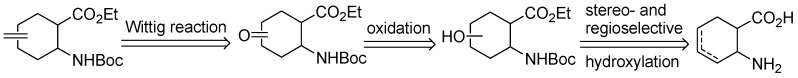
Retrosynthetic route to exomethylene cyclohexane β-amino esters.

A convenient and simple novel regio- and stereocontrolled synthetic procedure for the access to cyclohexane analogues of icofungipen is described, with an exomethylene group in different positions. Cyclohexene β-amino acids were subjected to regio- and stereoselective hydroxylation, oxidation and oxo-methylene interconversion as illustrated in the retrosynthetic scheme (Scheme 1).

**Scheme 2:**
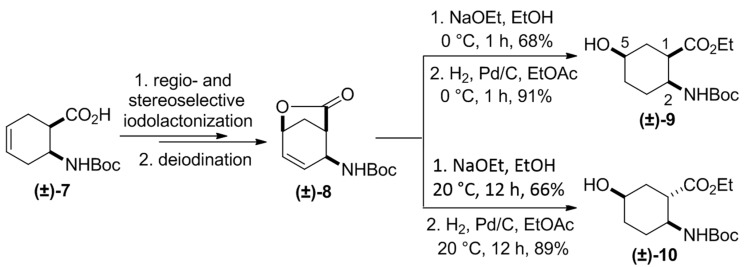
Synthesis of 5-hydroxylated β-amino ester diastereoisomers **(±)-9** and **(±)-10** [19].

The first synthetic approach was based on selective hydroxylation via iodolactonization. Racemic cyclohexene *cis*-β-amino acid **(±)-7** underwent regio- and stereoselective iodolactonization and deiodination by elimination to afford lactone **(±)-8**. Subsequent lactone opening in **(±)-8** with NaOEt at 0 °C for 1 h, followed by C-C double bond saturation, yielded 5-hydroxylated β-amino ester **(±)-9**. When the lactone opening with NaOEt was performed at 20 °C for 12 h, isomerization occurred with the participation of the active hydrogen at C-1, leading, after C=C reduction, to the thermodynamically more stable *trans* diastereoisomer **(±)-10** (Scheme 2) [19].

**Scheme 3:**
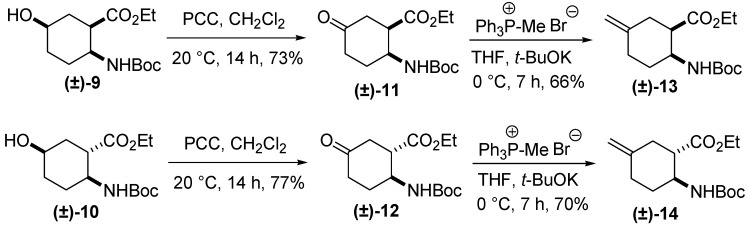
Synthesis of racemic exomethylene cyclohexane β-amino esters **(±)-13** and **(±)-14**.

By a modification of an earlier-described method, [3] oxidation of **(±)-9** and **(±)-10** with pyridinium chlorochromate (PCC) in CH_2_Cl_2_ at 20 °C afforded the corresponding oxo ester stereoisomers **(±)-11** and **(±)-12** [19]. Icofungipen analogues **(±)-13** and **(±)-14** were next synthesized from **(±)-11** and **(±)-12** via Wittig reactions by oxo-methylene exchange with the phosphorane generated from methyltriphenylphosponium bromide/*t*-BuOK at 0 °C (Scheme 3).

**Scheme 4:**
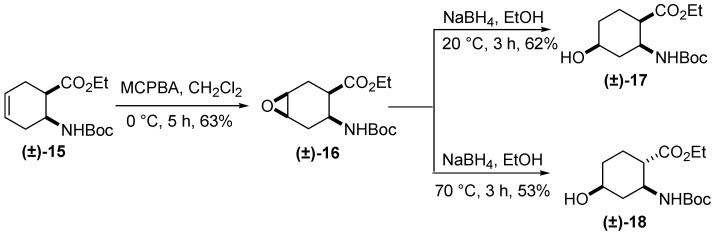
Synthesis of 4-hydroxylated β-amino ester diastereoisomers **(±)-17** and **(±)-18** [22].

The next synthetic approach to novel cyclohexane icofungipen analogues consisted in hydroxylation of the olefinic bond of the cyclohexene *cis*-β-amino ester **(±)-15** via *cis*-diastereoselective epoxidation with MCPBA and regioselective reductive oxirane opening with NaBH_4_, [20,21] with the hydride attack at C-5, resulting in the 4-hydroxylated β-amino ester diastereoisomers **(±)-17** and, at higher temperature, through isomerization at the active methyne **(±)-18** (Scheme 4) [22]. It may be noted that **(±)-18** was synthesized earlier in an alternative way from **(±)-15** [22].

**Scheme 5:**
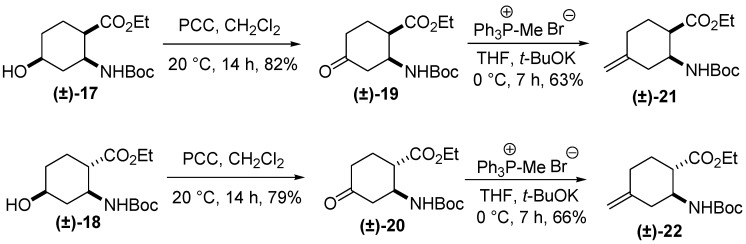
Synthesis of racemic exomethylene cyclohexane β-amino esters **(±)-21** and **(±)-22**.

Hydroxylated esters **(±)-17** and **(±)-18** were readily oxidized with PCC to oxo esters **(±)-19** and **(±)-20** [22]. Compounds **(±)-21** and **(±)-22**, with the methylene function at position 4, isomers of **(±)-13** and **(±)-14**, were readily prepared from **(±)-19** and **(±)-20** in Wittig reactions with the phosphorane generated *in situ* from methyltriphenylphosponium bromide/*t-*BuOK (Scheme 5).

**Scheme 6:**
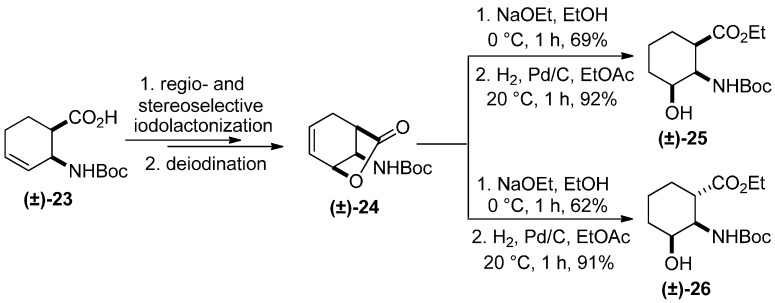
Synthesis of 3-hydroxylated β-amino ester stereoisomers **(±)-25** and **(±)-26** [6].

Other regio- and stereoisomers were synthesized by regio- and stereoselective iodolactonization and deiodination of β-aminocyclohex-3-enecarboxylic acid **(±)-23**, followed by selective lactone opening with NaOEt and hydrogenation of the amino lactone intermediate **(±)-24** to furnish analogously to **(±)-8** (Scheme 2) the 3-hydroxylated β-amino ester stereoisomers **(±)-25** and **(±)-26** (Scheme 6) [23].

**Scheme 7:**
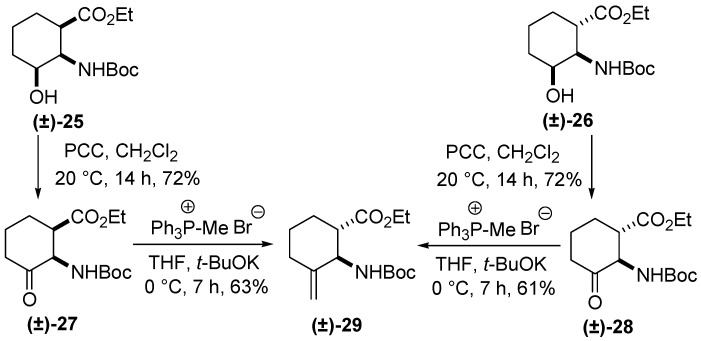
Synthesis of racemic **(±)-29**.

A modification of an earlier-described method [23] was next used: oxidation of hydroxylated amino esters **(±)-25** and **(±)-26** with PCC in CH_2_Cl_2_ at room temperature led to the corresponding *cis* and *trans* keto esters **(±)-27** and **(±)-28** [23]. Although *cis*-keto aminocarboxylate **(±)-27** afforded the Wittig product on treatment with methyltriphenylphosphonium bromide/*t*-BuOK in tetrahydrofurane due to the presence of the active hydrogen isomerization occurred at C-2 under alkaline conditions and gave the thermodynamically more stable **(±)-29** (only the relative stereochemistry is shown), in which the amino and carboxylate functions are in a *trans* relationship; *trans* amino ester **(±)-28** reacted with the phosphonium salt in the presence of *t-*BuOK to yield **(±)-29** stereoisomer with the ester and carbamate in the *trans* arrangement (Scheme 7).

**Scheme 8:**
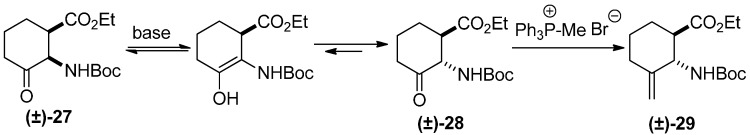
Formation of **(±)-29** from **(±)-27** through *trans-*amino ester **(±)-28**.

The isomerization of *cis*-**(±)-27** at C-2 during the Wittig reaction with methyltriphenylphosponium bromide/*t*-BuOK in THF to give **(±)-29** through *trans* amino ester **(±)-28** is depicted in Scheme 8.

**Scheme 9:**
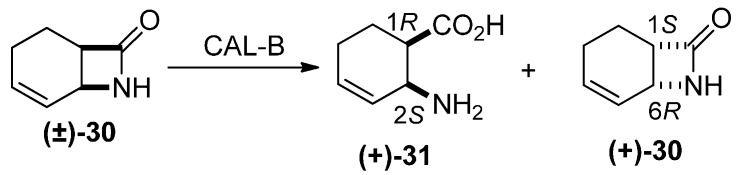
Synthesis of enantiomerically pure lactam **(+)-30**.

The above experiments (**27**→**29** and **28**→**29**) with the racemates led us to suppose that both enantiomers of **29** could be obtained by starting from an enantiomerically pure bicyclic lactam. For this purpose, therefore, enantiomerically pure β-lactam **(+)-30** (*ee* = 99%) was prepared by CAL-B-catalyzed ring-opening of racemic lactam **(±)-30** (Scheme 9) [24].

**Scheme 10:**
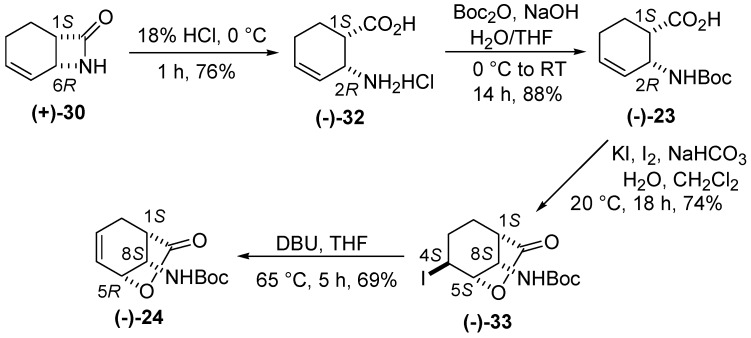
Synthesis of enantiomer lactone **(−)-24**.

Enantiomerically pure β-lactam **(+)-30** was next transformed by an earlier-published procedure [23] to the corresponding *N-*Boc amino acid, which was then converted to enantiopure bicyclic lactone **(−)-24** (Scheme 10).

**Scheme 11:**
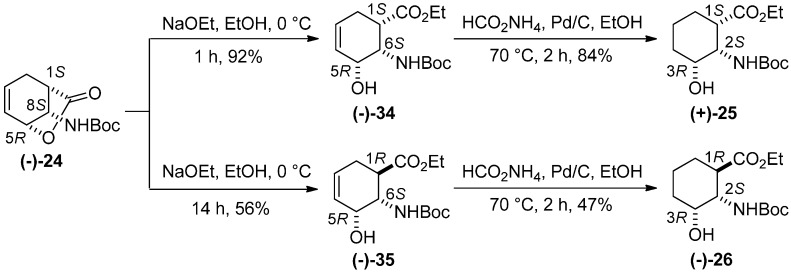
Synthesis of amino ester stereoisomers **(+)-25** and **(−)-26**.

On treatment with NaOEt at 0 °C, optically pure lactone **(−)-24** gave the *all-cis* 3-hydroxylated β-amino ester **(−)-34**, [23] whereas at room temperature for 14 h isomerization at C-1 led to **(−)-35** [23]. On catalytic hydrogenation, these compounds afforded hydroxylated cyclohexane β-amino esters **(+)-25** and **(−)-26**, [6] respectively, in enantiomerically pure form (Scheme 11) [23].

Analogously to the racemates, **(+)-25** and **(−)-26** [23] underwent oxidation to the corresponding enantiopure *cis* and *trans* **(+)-27** and **(−)-28** (*ee* = 99%).

**Scheme 12:**
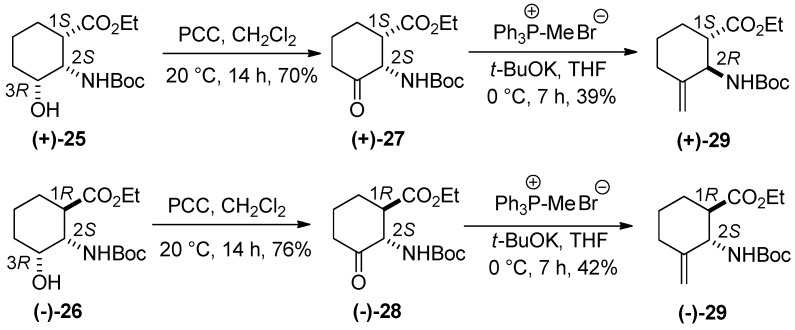
Synthesis of amino ester enantiomers **(+)-29** and **(−)-29**.

On reaction with phosphorane generated *in situ* from methyltriphenyphosphonium bromide/*t*-BuOK, **(+)-27** participated in isomerization at C-2 to give the thermodynamically more stable **(+)-29** (*ee* = 90.6%), while under similar conditions **(−)-28** yielded its opposite enantiomer **(−)-29** (*ee* = 86.6%). The chiral centers at C-1 or C-2 in **(+)-27** may theoretically both be affected (both active hydrogens) but this was not observed. Only C-2 underwent isomerization, leading to the thermodynamically more stable derivative **(+)-29** with the carbamate and ester groups in a *trans* relative relationship. (Scheme 12).

## 3. Experimental Section

### 3.1. General Procedure for the Methylenation of Oxo Esters

To a solution of methyltriphenylphosphonium bromide (2 mmol) in THF (15 mL), *t-*BuOK (1 equiv.) was added and the solution was stirred for 15 min at 20 °C. The β-aminooxocarboxylate (1 equiv.) was then added and the mixture was further stirred at this temperature. After 6 h, water (15 mL) was added, and the mixture was extracted with CH_2_Cl_2_ (2 × 15 mL). The organic layer was dried (Na_2_SO_4_) and concentrated, and the crude product was purified by column chromatography on silica gel (*n*-hexane/EtOAc 9:1).

Ethyl (1R*,2S*)-2-(tert-butoxycarbonylamino)-5-methylenecyclohexanecarboxylate [**(±)-13**]. A colorless oil, yield: 66%. R_f_ = 0.65 (*n*-hexane/EtOAc 4:1); ^1^H-NMR (CDCl_3_, 400 MHz): δ = 1.22 (t, 3H, CH_3_, *J* = 7.00 Hz), 1.41 (s, 9H, *t-*Bu), 1.71–1.80 (m, 1H, CH_2_), 1.83–1.90 (m, 1H, CH_2_), 2.09–21 (m, 1H, CH_2_), 2.23–2.30 (m, 1H, CH_2_), 2.32–2.38 (m, 1H, CH_2_), 2.57–2.63 (m, 1H, CH_2_), 2.82–2.88 (m, 1H, H-1), 3.96–4.02 (m, 1H, H-2), 4.07–4.20 (m, 2H, OCH_2_), 4.63–4.70 (m, 2H, CH_2_), 5.38 (brs, 1H, N-H). ^13^C-NMR (DMSO, 100 MHz): δ = 14.9, 29.1, 30.1, 32.3, 32.8, 46.8, 47.9, 60.6, 78.6, 109.4, 147.1, 158.0, 173.0. Anal. Calcd for C_15_H_25_NO_4_: C 63.58, H 8.89, N 4.94; found: C 63.20, H 8.61, N 4.68.

Ethyl (1S*,2S*)-2-(tert-butoxycarbonylamino)-5-methylenecyclohexanecarboxylate [**(±)-14**]. A white solid, mp 102–103 °C; yield: 70%. R_f_ = 0.6 (*n*-hexane/EtOAc 4:1); ^1^H-NMR (CDCl_3_, 400 MHz): δ = 1.21 (t, 3H, CH_3_, *J* = 7.00 Hz), 1.41 (s, 9H, *t-*Bu), 2.06–2.19 (m, 1H, CH_2_), 2.24–2.43 (m, 5H, CH_2_, H-1), 3.79–3.86 (m, 1H, H-2), 4.11–4.20 (m, 2H, OCH_2_), 4.42 (brs, 1H, N-H), 4.70–4.73 (m, 2H, CH_2_). ^13^C-NMR (DMSO, 100 MHz): δ = 14.9, 29.1, 33.3, 33.6, 36.7, 50.3, 50.9, 60.7, 78.3, 110.2, 146.0, 156.0, 173.7. Anal. Calcd for C_15_H_25_NO_4_: C 63.58, H 8.89, N 4.94; found: C 63.22, H 9.11, N 4.69.

Ethyl (1R*,2S*)-2-(tert-butoxycarbonylamino)-4-methylenecyclohexanecarboxylate [**(±)-21**]. A white solid, mp 56–58 °C; yield: 63%. R_f_ = 0.6 (*n*-hexane/EtOAc 4:1); ^1^H-NMR (CDCl_3_, 400 MHz): δ = 1.16 (t, 3H, CH_3_, *J* = 7.10 Hz), 1.43 (s, 9H, *t*-Bu), 1.79–1.86 (m, 1H, CH_2_), 1.88–2.03 (m, 1H, CH_2_), 2.11–2.19 (m, 1H, CH_2_), 1.23–1.33 (m, 1H, CH_2_), 2.38–2.45 (m, 2H, CH_2_), 2.77–2.82 (m, 1H, H-1), 4.09–4.23 (m, 3H, OCH_2_, H-2), 4.78–4.80 (m, 1H, =CH), 4. 83–4.86 (m, 1H, =CH), 5.06 (brs, 1H, N-H). ^13^C-NMR (CDCl_3_, 100 MHz): δ = 14.6, 26.4, 28.8, 32.5, 39.8, 45.4, 49.7, 60.9, 79.6, 111.4, 144.5, 155.4, 173.7. Anal. Calcd for C_15_H_25_NO_4_: C 63.58, H 8.89, N 4.94; found: C 63.23, H 8.60, N 4.68.

Ethyl (1S*,2S*)-2-(tert-butoxycarbonylamino)-4-methylenecyclohexanecarboxylate [**(±)-22**]. A white solid, mp 99–101 °C; yield: 66%. R_f_ = 0.55 (*n*-hexane/EtOAc 4:1); ^1^H-NMR (CDCl_3_, 400 MHz): δ = 1.21 (t, 3H, CH_3_, *J* = 7.10 Hz), 1.41 (s, 9H, *t*-Bu), 1.78–1.88 (m, 1H, CH_2_), 1.89–1.98 (m, 1H, CH_2_), 2.00–2.10 (m, 2H, CH_2_), 2.29–2.38 (m, 1H, CH_2_), 2.56–2.61 (m, 1H, CH_2_), 2.62–2.69 (m, 1H, H-1), 3.83–3.96 (m, 1H, H-2), 4.17–4.24 (m, 2H, OCH_2_), 4.60 (brs, 1H, N-H), 5.79–5.82 (m, 2H, =CH), ^13^C-NMR (DMSO, 100 MHz): δ = 14.6, 27.8, 28.8, 32.8, 40.5, 48.2, 61.0, 78.0, 110.9, 144.9, 152.0, 173.8. Anal. Calcd for C_15_H_25_NO_4_: C 63.58, H 8.89, N 4.94; found: C 63.78, H 8.66, N 5.23.

Ethyl (1S*,2R*)-2-(tert-butoxycarbonylamino)-3-methylenecyclohexanecarboxylate [**(±)-29**]. A white solid, mp 75–77 °C; yield: 63%. R_f_ = 0.65 (*n*-hexane/EtOAc 4:1); ^1^H-NMR (CDCl_3_, 400 MHz): δ = 1.22 (t, 3H, CH_3_, *J* = 7.10 Hz), 1.22–1.30 (m, 1H, CH_2_) 1.40 (s, 9H, t-Bu), 1.75–1.84 (m, 2H, CH_2_), 1.95–2.02 (m, 1H, CH_2_), 2.07–2.19 (m, 1H, CH_2_), 2.21–2.28 (m, 1H, CH_2_), 2.39–2.43 (m, 1H, H-1), 4.11–4.20 (m, 2H, OCH_2_), 4.24–4.35 (m, 1H, H-2), 4.39 (brs, 1H, N-H), 4.79–4.83 (m, 2H, CH_2_). ^13^C-NMR (DMSO, 100 MHz): δ = 14.9, 26.6, 29.1, 29.7, 34.8, 50.7, 55.0, 60.6, 78.3, 107.7, 147.9, 155.7, 173.9. Anal. Calcd for C_15_H_25_NO_4_: C 63.58, H 8.89, N 4.94; found: C 63.80, H 8.60, N 5.22.

### 3.2. Characterization of the Enantiomerically Pure Substances

The *ee* values for **(+)-27** and **(−)-28** were determined on a HPLC (ChiralPak IA, Chiral Technologies Europe, Illkirch-Graffenstaden, France) 5 μ column (0.4 cm × 1 cm): for **(+)-27** (*ee* 99%), mobile phase: *n*-hexane/2-propanol (80/20); flow rate 0.5 mL/min; detection at 205 nm; retention time (min): 11.14 (for antipode: 10.68); for **(−)-28** (*ee* 99%), mobile phase: *n*-hexane/2-propanol (70/30); flow rate 0.5 mL/min; detection at 205 nm; retention time (min): 11.8 (for antipode: 25.1).

The *ee* values for **(−)-29** and **(+)-29** were determined on a HPLC (ChiralPak IA) 5 μ column (0.4 cm × 1 cm), for **(−)-29** (*ee* 90%): mobile phase: *n*-hexane/2-propanol (70/30); flow rate 0.5 mL/min; detection at 205 nm; retention time (min): 9.25; for **(+)-29** (*ee* 86%): mobile phase: *n*-hexane/2-propanol (70/30); flow rate 0.5 mL/min; detection at 205 nm; retention time (min): 10.36.

All ^1^H-NMR spectra recorded for the enantiomeric substances were the same as for the corresponding racemic counterparts.

*(1S,2R)-2-Aminocyclohex-3-enecarboxylic acid hydrochloride* [**(−)-32**] [20,21]. A white solid; mp 203–206 °C; yield: 76%. ${\left[\alpha \right]}_{D}^{25}$ = −89.5 (*c* 0.335, EtOH).

*(1S,2R)-2-(tert-Butoxycarbonyl)cyclohex-3-enecarboxylic acid* **[(−)-23**]. A white solid; mp 115–118 °C; yield: 88%. ${\left[\alpha \right]}_{D}^{25}$ = −26.6 (*c* 0.315, EtOH), (for the opposite enantiomer see reference [23]).

*tert-Butyl (1S,4S,5S,8S)-4-iodo-7-oxo-6-oxabicyclo[3.2.1]octan-8-ylcarbamate* [**(−)-33**]. A white solid; mp 50–53 °C; yield: 74%. ${\left[\alpha \right]}_{D}^{25}$ = −54.4 (*c* 1.9, EtOH) (for the opposite enantiomer, see reference [23]).

*tert-Butyl (1S,5R,8S)-7-oxo-6-oxabicyclo[3.2.1]oct-3-en-8-ylcarbamate* [**(−)-24**]. A white solid; mp 157–159 °C; yield: 69%. ${\left[\alpha \right]}_{D}^{25}$ = −107.6 (*c* 0.35, EtOH) (for the opposite enantiomer, see reference [23]).

*Ethyl (1S,5R,6S)-6-(tert-butoxycarbonyl)-5-hydroxycyclohex-3-enecarboxylate* [**(−)-34**]. A colorless oil; yield: 92%. ${\left[\alpha \right]}_{D}^{25}$ = −21.6 (*c* 0.375, EtOH) (for the opposite enantiomer, see reference [23]).

*Ethyl (1R,5R,6S)-6-(tert-butoxycarbonyl)-5-hydroxycyclohex-3-enecarboxylate* [**(−)-35**]. A colorless oil; yield: 56%. ${\left[\alpha \right]}_{D}^{25}$ = −79.6 (*c* 0.48, EtOH) (for the opposite enantiomer, see reference [23]).

*Ethyl (1S,2S,3R)-2-(tert-butoxycarbonyl)-3-hydroxycyclohexanecarboxylate* [**(+)-25**]. A white solid; mp 76–79 °C; yield: 87%. ${\left[\alpha \right]}_{D}^{25}$ = +24.6 (*c* 0.62, EtOH).

*Ethyl (1R,2S,3R)-2-(tert-butoxycarbonyl)-3-hydroxycyclohexanecarboxylate* [**(−)-26**]. A white solid; mp 92–94 °C; Yield: 47%. ${\left[\alpha \right]}_{D}^{25}$ = −38.4 (*c* 0.61, EtOH), (for the opposite enantiomer see reference [23]).

*Ethyl (1S,2S)-2-(tert-butoxycarbonyl)-3-oxocyclohexanecarboxylate* [**(+)-27**]. A colorless oil; yield: 70%. ${\left[\alpha \right]}_{D}^{25}$ = +54.3 (*c* 0.415, EtOH), (for the racemic compound, see reference [23]).

*Ethyl (1R,2S)-2-(tert-butoxycarbonyl)-3-oxocyclohexanecarboxylate* [**(−)-28**]. A white solid; mp 85–88 °C; yield: 76%. ${\left[\alpha \right]}_{D}^{25}$ = −12.7 (*c* 0.53, EtOH) (for the racemic compound see reference [23]).

*Ethyl (1S,2R)-2-(tert-butoxycarbonyl)-3-methylenecyclohexanecarboxylate* [**(+)-29**]. A colorless oil; yield: 39%. ${\left[\alpha \right]}_{D}^{25}$ = +25.8 (*c* 0.38, EtOH); *ee* = 90.6%.

*Ethyl (1R,2S)-2-(tert-butoxycarbonyl)-3-methylenecyclohexanecarboxylate* [**(−)-29**]. A colorless oil; yield: 42%. ${\left[\alpha \right]}_{D}^{25}$ = −14.1 (*c* 0.33, EtOH); *ee* = 86.7%.

## 4. Conclusions

Cyclohexane β-amino esters with an extracyclic methylene at position 3, 4 or 5 have been regio- and stereoselectively synthetized from 2-aminocyclohexenecarboxylic acid regioisomers by transformation of the ring olefinic bond via three different regio- and stereocontrolled hydroxylation procedures, followed by deoxygenation through oxo-methylene interconversion via Wittig reactions. An enantiodivergent route starting from a bicyclic β-lactam enantiomer permitted the synthesis of both enantiomers of a cyclohexane icofungipen analogue.
